# Molecular and Clinical Characterization of UBE2S in Glioma as a Biomarker for Poor Prognosis and Resistance to Chemo-Radiotherapy

**DOI:** 10.3389/fonc.2021.640910

**Published:** 2021-05-27

**Authors:** Li Hu, Xingbo Cheng, Zev Binder, Zhibin Han, Yibo Yin, Donald M. O’Rourke, Sida Wang, Yumeng Feng, Changjiang Weng, Anhua Wu, Zhiguo Lin

**Affiliations:** ^1^ Department of Neurosurgery, The First Affiliated Hospital of Harbin Medical University, Harbin, China; ^2^ Department of Neurosurgery, Perelman School of Medicine at the University of Pennsylvania, Philadelphia, PA, United States; ^3^ State Key Laboratory of Veterinary Biotechnology, Harbin Veterinary Research Institute of Chinese Academy of Agricultural Sciences, Harbin, China; ^4^ Department of Neurosurgery, The First Hospital of China Medical University, Shenyang, China

**Keywords:** glioblastoma multiform, UBE2S, PTEN, pAkt, chemo-radiotherapy, prognosis

## Abstract

Glioblastoma is the most common and lethal brain cancer globally. Clinically, this cancer has heterogenous molecular and clinical characteristics. Studies have shown that UBE2S is highly expressed in many cancers. But its expression profile in glioma, and the correlation with clinical outcomes is unknown. RNA sequencing data of glioma samples was downloaded from the Chinese Glioma Genome Atlas and The Cancer Genome Atlas. A total of 114 cases of glioma tissue samples (WHO grades II-IV) were used to conduct protein expression assays. The molecular and biological characteristics of UBE2S, and its prognostic value were analyzed. The results showed that high UBE2S expression was associated with a higher grade of glioma and PTEN mutations. In addition, UBE2S affected the degree of malignancy of glioma and the development of chemo-radiotherapy resistance. It was also found to be an independent predictor of worse survival of LGG patients. Furthermore, we identified five UBE2S ubiquitination sites and found that UBE2S was associated with Akt phosphorylation in malignant glioblastoma. The results also revealed that UBE2S expression was negatively correlated with 1p19q loss and IDH1 mutation; positively correlated with epidermal growth factor receptor amplification and PTEN mutation. This study demonstrates that UBE2S expression strongly correlates with glioma malignancy and resistance to chemo-radiotherapy. It is also a crucial biomarker of poor prognosis.

## Introduction

GBM is the most common primary malignant brain tumor in adults (1). Despite advances in comprehensive therapy, such as development of neurosurgical resection, adjuvant radiotherapy, and alkylating agent temozolomide (TMZ) chemotherapy, GBM patients are characterized by poor prognosis and the 5-year survival rate is below 10%. The median overall survival is 12 to 15 months following primary diagnosis (2, 3), due to aggressiveness of the tumor, resistance to treatments, and recurrence over time (4). Advances in gene technology have enabled identification of molecular signatures for classification of glioma in recent years. Previous studies report that a complicated molecular network of signaling pathways is implicated in mediating malignancy of glioma (5). Therefore, exploring such therapeutic targets that are capable of regulating multiple molecules or signaling pathways in progression of glioma and TMZ resistance are beneficial for improving glioma treatment. Recent studies explored the role of genetic mutations in glioma, however, it is not clear which mutation is significantly associated with specific characteristics of glioma. Classification based only on histopathology does not effectively describe all the malignant features of GBMs, mainly their responses to treatments. For example, some GBMs are mainly sensitive to radio-chemotherapy whereas others are resistant to radiotherapy and chemotherapy. Notably, GBMs are aggressive and are characterized by early recurrence, whereas others progress slowly for prolonged period (6, 7). Molecular parameters are now considered for GBMs classification due to these limitations in traditional classification methods.

UBE2S, a 24 kDa ubiquitin-conjugating enzyme, is highly expressed in most human cancers, including breast cancer, colon tumors, and ovarian cancer (8–11), compared with normal tissues, implying that it is involved in oncogenesis. Moreover, UBE2S is highly expressed in hepatocellular carcinoma (HCC), where it interacts with TRIM28 and enhances ubiquitination of p27, thus promoting HCC progression (12). UBE2S associates with and targets pVHL for ubiquitin-mediated proteolysis, thus stabilizing HIF-1α. Furthermore, overexpression of UBE2S promotes proliferation, invasion and metastasis through pVHL-HIF pathway (9). UBE2S knockdown results in reduction in FAK phosphorylation at Tyr397, thus inhibiting signal level for cell migration and invasion (13). Akt1 phosphorylates UBE2S and enhances its stability, and knockdown of UBE2S expression inhibits NHEJ-mediated DNA repair, however, this activity has not been validated using clinical data.

Large scale meta-analysis of cancer microarray data show that UBE2S is a commonly activated gene in multiple cancers. However, its expression in glioma tissues and the potential correlation between UBE2S and clinical outcome in patients with glioma have not been explored. The aim of this study was to explore expression levels of UBE2S in glioma, and to explore the relationship between UBE2S expression and prognosis of glioma, and chemo-radiotherapy resistance.

## Materials and Methods

### Clinical Data and Tumor Specimen Selection

A retrospective cohort of 114 glioma patients was included in the present study. Glioma samples were obtained from open-craniotomy surgery performed at the First Affiliated Hospital of Harbin Medical University. Inclusion criteria were as follows: primary diagnosis of glioma between 2008 and 2016 and no previous diagnosis of carcinoma. Patients who had received neoadjuvant treatment before primary surgery were excluded. Normal brain specimens (n = 5) were obtained from patients with severe brain injury who underwent partial normal brain resection and decompression. Histopathological analysis (according to the WHO classification) were performed by two independent neuropathologists, and a consensus was reached in all cases. Ethical approval for carrying out this study was obtained from the Committees for the Ethical Review of Research at the First Affiliated Hospital of Harbin Medical University, Harbin, China. Informed consent was obtained from each participant before participation in this study. mRNA expression microarray data and related clinical information for glioma samples were retrieved from The Cancer Genome Atlas and Chinese Glioma Genome Atlas databases.

### Cell lines, Antibodies, and Reagents

Glioma cell lines were obtained from Helen Diller Cancer Center of the University of California, San Francisco (UCSF). UBE2S-specific shRNA (5’-GGGCTCTCTTCCTCCTTCCAC-3’) and control shRNA (5’-TTCTCCGAACGTGTCACGT-3’) oligonucleotides were established. Knockdown of endogenous UBE2S expressions in U87 and U251 cells was performed using lentivirus-expressing shRNA as described previously (14). Rabbit anti-GAPDH and anti-Ki67 polyclonal antibodies were purchased from Proteintech Group (Proteintech Group, Chicago, IL). Rabbit anti-HA and anti-Flag polyclonal antibodies were purchased from Sigma-Aldrich (St. Louis, MO). Rabbit anti-pAkt S473, and anti-PTEN polyclonal antibodies were purchased from Abcam (Cambridge, MA, USA). Anti-Flag M2 magnetic Beads were purchased from Sigma-Aldrich (St. Louis, MO), whereas MK-2206 was purchased from Selleck Chemicals (Houston, TX, USA).

### Immunohistochemistry

Paraffin-embedded sections (4μm) of excised specimens were immunostained for determination of UBE2S protein level. Staining was performed using the streptavidin-biotin peroxidase complex method, following the manufacturer’s instructions (Dako, Denmark). Negative and positive controls were included in the immunohistochemistry procedure. Immunohistochemical staining score standard: positive rate of UBE2S staining more than 10% was considered as positive group. UBE2S staining of glioma was divided into positive group and negative group based on this criterion (15). All immunostained sections were reviewed by two investigators who were blinded to the treatments and the immunostained images were acquired using an Olympus inverted microscope.

### Immunoprecipitation and Immunoblotting

Tissue were harvested for total protein extraction. Protein concentration was determined using Bradford method. For immunoprecipitation, lysates were incubated with anti-Flag M2 magnetic beads overnight at 4°C. Immunoprecipitants were then subjected to electrophoresis. For western blot analysis, equal amounts of protein were loaded and separated by SDS-PAGE. Gels were equilibrated in transfer buffer (50 mM Tris, 40 mM glycine, 0.375% SDS and 20% methanol) and transferred to a PVDF membrane (Millipore, USA) through electrophoresis. The membrane was blocked with 5% skim milk in TBST buffer (20 mM Tris-HCl, pH 7.4, 150 mM NaCl and 0.1% Tween20) and incubated over night at 4°C with specific primary antibodies. The membrane was then washed with TBST, and incubated with horseradish peroxidase conjugated secondary antibodies (Invitrogen, USA) for 1 h (16).

### Gene Ontology (GO) and Kyoto Encyclopedia of Genes and Genomes (KEGG) Analysis

To identify the biological processes and KEGG signaling pathways related to UBE2S expression in glioma, GO enrichment analysis and KEGG pathway analysis were performed using clusterProfler to explore the biological significance and key pathways associated with UBE2S of differentially expressed genes (DEGs) (criteria: p-value < 0.05, significantly enriched). Fisher’s exact test was used to identify significant GO terms and pathways.

### Apoptosis Assays by Annexin V-FITC/PI Double Staining

U87 or U251 cells were seeded in 6-well plates at a density of 1×10^5^ cells/well. After treatment with 6MV X-Ray (10Gy), cells were collected and stained using an Annexin V-FITC/PI Apoptosis Detection kit according to the manufacturer’s instructions (no. C1062, Beyotime, Nanjing, China). Cells were then analyzed using a flow cytometer (Beckhman Coulter Inc, Brea, CA, USA).

### Statistical Analysis

Overall survival analysis was performed using Log-rank test. Cox regression analysis was performed using the survival package in R software. Statistical differences among groups were determined by one-way analysis of variance (ANOVA) followed by Newman-Keuls test for multiple comparisons. In those experiments where experimental values were normalized to controls, statistical difference compared with the controls was calculated using Kruskal-Wallis test or Wilcoxon test. Correlations between UBE2S expression and various clinicopathological characteristics were assessed by χ2-tests. SPSS 17.0, Prism5 and R software (version 3.3.2) were used for statistical analysis. p<0.05 was considered as statistically significant. Significance was set at *p<0.05, **p<0.01 or ***p<0.001.

## Results

### UBE2S Expression Is Associated With Glioma Grades

**Figure 1:**
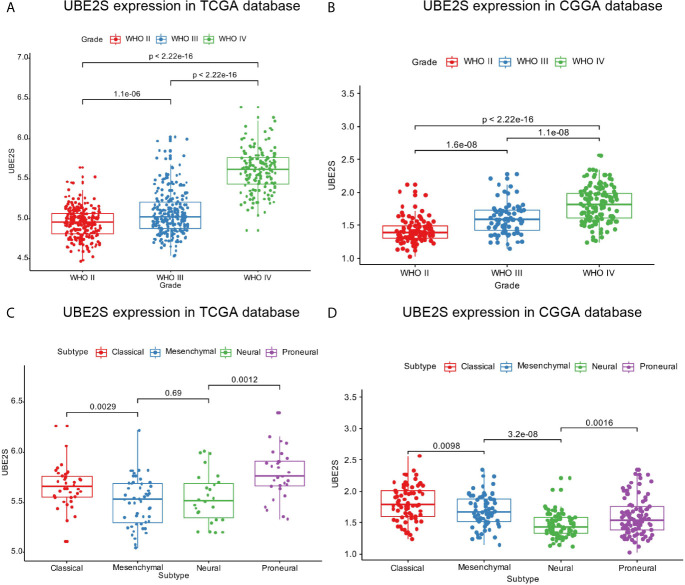
UBE2S was significantly upregulated in glioblastoma. **(A, B)** UBE2S expression level was significantly high in glioblastoma (WHO IV) samples in TCGA and CGGA data set. **(C, D)** UBE2S expression pattern in different molecular subtypes in TCGA and CGGA data set. P is based on Kruskal-Wallis test.

Expression pattern of UBE2S across grades and subtypes defined by expression clusters by the TCGA work-group were explored (17). In the TCGA cohort, UBE2S expression increased with increasing grade of glioma with the highest expression level in WHO Grade IV (p < 0.0001, **Figure 1A**). Similar findings were obtained with the CGGA cohort (**Figure 1B**) which showed that UBE2S expression was highly correlated with the malignancy of glioma. Analysis based on the TCGA subtype classification system showed that the Proneural subtype had the highest UBE2S expression level whereas the mesenchymal subtype had the lowest UBE2S expression in TCGA cohort (**Figure 1C**). In the CGGA cohort, UBE2S expression was up-regulated in Classical subtypes (**Figure 1D**).

### UBE2S-Related Genomic Alterations and Biological Processes

**Figure 2:**
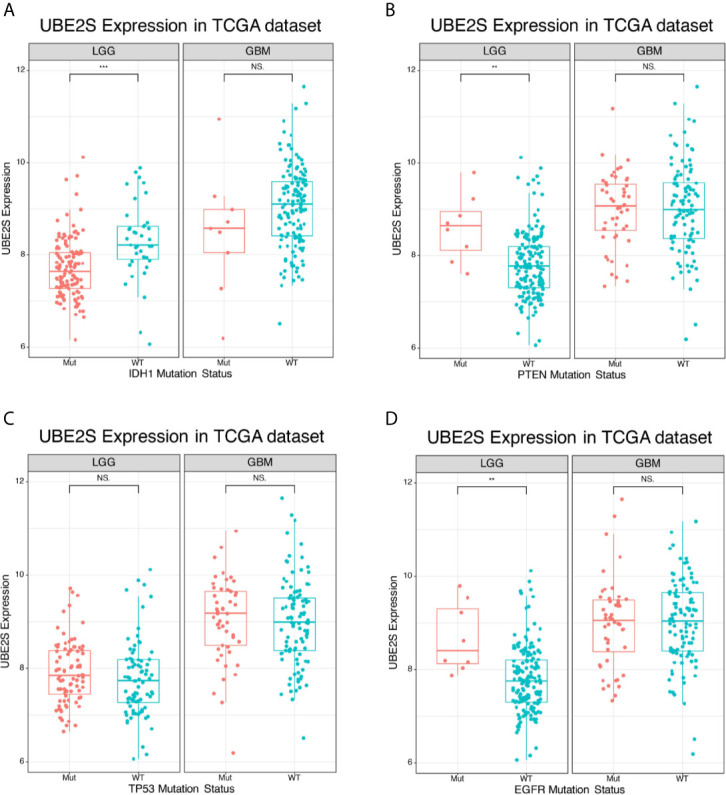
Expression of UBE2S in glioma of different mutation types. **(A)** UBE2S was significantly upregulated in IDH1 wild-type LGG glioma in TCGA data set compared with the mutants. **(B)** UBE2S was significantly upregulated in PTEN mutation LGG glioma in TCGA data set compared with the wild-type. **(C)** UBE2S expression was not significantly different between TP53 mutation and TP53 wild-type LGG/GBM glioma in TCGA data set. **(D)** UBE2S was significantly upregulated in EGFR mutation LGG glioma in TCGA data set. Wilcoxon test was used to determine statistical difference. ***P < 0.001, **P < 0.01, NS represents non-significant.

IDH1 mutation status is a clinically effective molecular marker in glioma (18). Glioma patients were divided into IDH1 mutation group and IDH1 wild-type group and the association between UBE2S expression level and the IDH1 status was determined. In the TCGA cohort, UBE2S expression level was significantly higher in the IDH1 wild-type group compared with expression level in the IDH1 mutation group of LGG patients (**Figure 2A**). However, no statistical significance was detected between the two groups of GBM patients. Further analysis of the relationship between UBE2S expression level and PTEN status showed that UBE2S expression level was significantly higher in PTEN mutation group compared with the expression level in the PTEN wild-type group of LGG patients (**Figure 2B**). However, no statistical significance was observed between the two groups of GBM patients (**Figure 2B**). Glioma patients were grouped into TP53 wild-type group and TP53 mutation group, and analysis showed no significant difference in UBE2S expression between the two groups (**Figure 2C**). Analysis of the relationship between UBE2S expression level and EGFR status, showed that UBE2S expression was significantly higher in EGFR mutation group compared with the level in EGFR wild-type group of LGG patients (**Figure 2D**). However, no statistical significance was observed between the two groups in GBM patients (**Figure 2D**). These findings show that UBE2S has a PTEN mutant, EGFR mutant and IDH1 wild-type preference in LGG patients.

**Figure 3:**
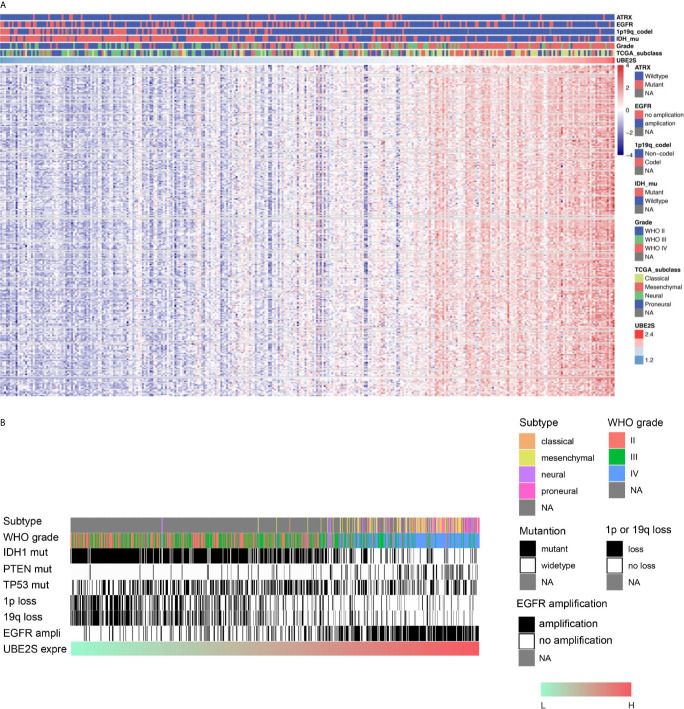
Correlations between UBE2S expression level with the classical genomic or transcriptional alterations in glioma. **(A)** Correlations between UBE2S expression level and the classical genomic or transcriptional alterations in whole grade glioma in CGGA data set. **(B)** Correlations of UBE2S expression level and the classical genomic or transcriptional alterations in whole grade glioma in TCGA data set. Pearson correlation analyses were used.

To explore the biological features of glioma associated with different UBE2S expression, Pearson correlation analyses were performed to determine the correlation between UBE2S expression and other genes in CGGA sequencing dataset. A total of 283 genes were correlated with UBE2S mRNA expression in the CGGA RNAseq dataset (|R| ≥ 0.6, p ≤ 0.01, **Figure 3A**). Further analysis on the oncogenic features of UBE2S showed that the genomic or transcriptional alterations associated with the origin or progression of glioma correlated with degrees of UBE2S expression level (**Figures 3A, B**). Selected glioma genes with high and low UBE2S expression level were separately derived from the TCGA RNA-sequencing database and a heatmap for enrichment scores was generated using the “pheatmap” package in R. IDH1-mutant lower grade glioma had significantly lower levels of UBE2S expression compared with the wild-type (**Figure 3A**). Co-deletion of 1p19q, which is an indicator of optimistic outcome, was mainly observed in glioma with lower UBE2S expression level. The incidence of malignant factor, EGFR amplification and PTEN mutation, was significantly higher in glioma samples with higher expression levels of UBE2S compared with those with low expression levels. On the other hand, TP53 mutation showed no association with UBE2S expression. Similar findings were observed in the TCGA cohort (**Figure 3B**) whereby UBE2S expression was significantly correlated with the key molecular characteristics of glioma.

The correlated genes were used for functional annotation analysis and were ranked by p value in increasing order. Analysis showed that UBE2S related genes were mainly involved in normal biological processes, such as ATPase activity, microtubule binding, tubulin binding, catalytic activity, acting on DNA, helicase activity in molecular function (**Figure S1A**); organelle fission, nuclear division, chromosome segregation, mitotic nuclear division, nuclear chromosome segregation in biological processes term (**Figure S1B**); chromosomal region, spindle, condensed chromosome, chromosome centromeric region in cell component (**Figure S1C**). Furthermore, KEGG pathways analyses were performed for comprehensive analysis of critical pathways related to UBE2S (**Figure S1D**). The most vital pathways of UBE2S included cell cycle, spliceosome, DNA replication, progesterone-mediated oocyte maturation, oocyte meiosis, cellular senescence, mismatch repair, nucleotide excision repair, base excision repair and fanconi anemia pathway (**Figure S1D**).

### Correlation Between UBE2S, Glioma Malignancy, and Chemo-Radiotherapy Resistance

**Figure 4:**
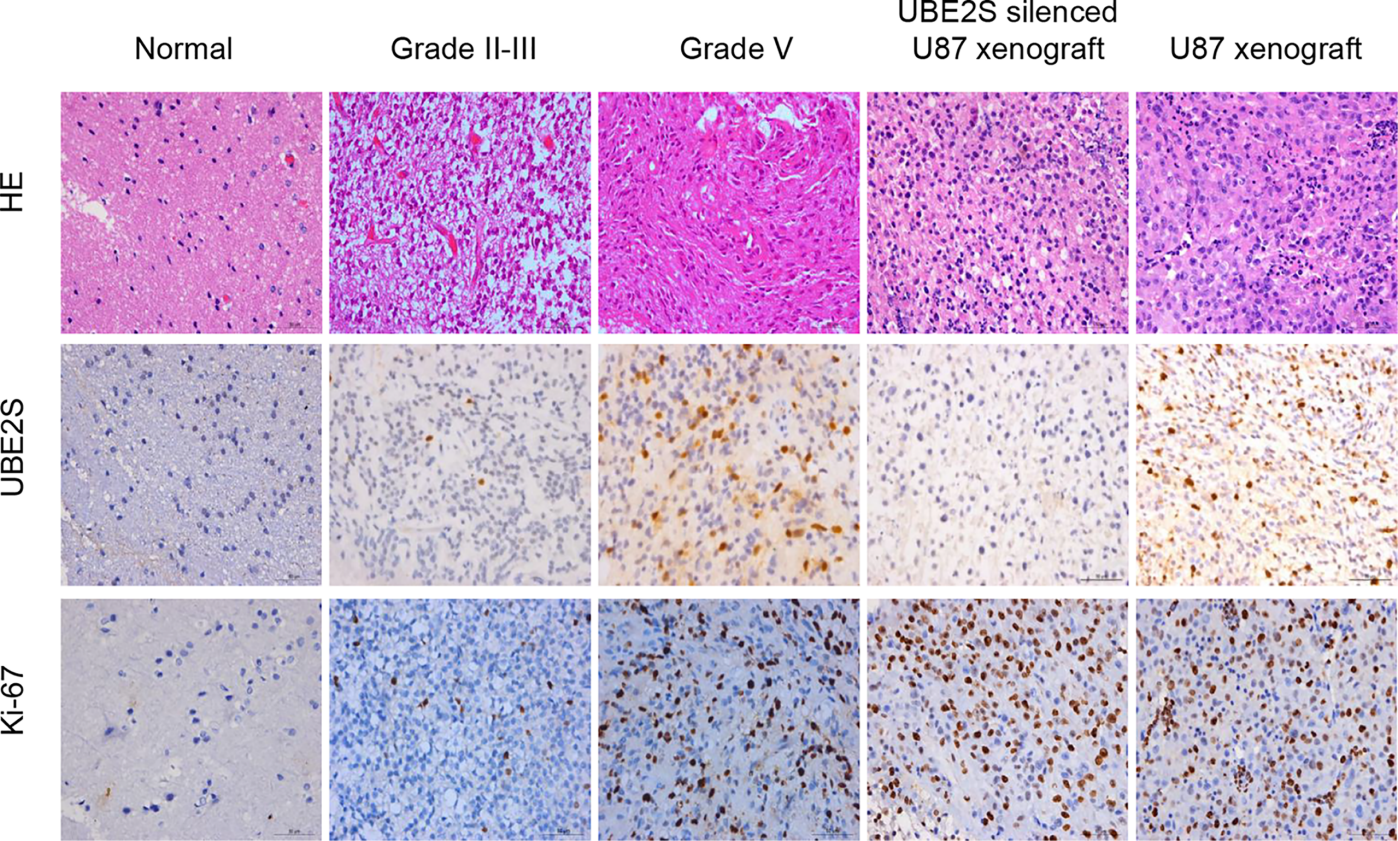
UBE2S was highly expressed in glioma tissue specimens. Representative images of hematoxylin/eosin (HE) staining showing UBE2S and Ki-67 immunoreactivity from the tissue. Tumor xenograft obtained from U87 cells was stained with anti-UBE2S monoclonal antibody and used as positive control (PC). Normal tissue and tumor xenograft from UBE2S-silenced U87 cells were stained with anti-UBE2S monoclonal antibody and used as negative control (NC) (×400). Scale bar: 50 μm.

To validate the RNA-based results, protein levels of UBE2S were determined by IHC in an independent cohort of human glioma (n = 114) and normal brain tissue (n = 5) from The First Affiliated Hospital of Harbin Medical University (Harbin, China). We found that UBE2S expression level was higher in grades III-IV glioma tumors (malignant glioma) compared with non-glioma brain tissue specimens or in grade II glioma tumors (**Figure 4**). These findings show that UBE2S protein expression was positively correlated with malignancy of glioma.

Baseline clinical characteristics of patients included in this study are shown in **Table 1**. Out of 114 glioma specimens 58 (51%) showed significant UBE2S expression. In addition, 61 out of the 114 patients (54%) responded poorly to neoadjuvant radio- or chemotherapy. Notably, samples from 53 out of the 61 poor responders (87%) showed positive staining for UBE2S. Further analysis showed that patients with positive staining for UBE2S were significantly associated with resistance to neoadjuvant therapy (p < 0.001). Subsequently, the correlation between UBE2S expression and chemo-radiotherapy sensitivity was determined using a Cox proportional hazards regression model. Univariate analysis showed a statistically significant correlation between chemo-radiotherapy sensitivity and expression levels of UBE2S (p < 0.001; hazard ratio HR = 0.016) (**Table S1**). Multivariate analysis further showed that expression level of UBE2S was an independent and significant predictor of chemo-radiotherapy sensitivity (p < 0.001; hazard ratio HR = 0.012) (**Table S1**).

**Table 1:** Clinicopathological characteristics of glioma patients for immunohistochemical investigation.

Characteristic	IHC-UBE2S (-) N (%)	IHC-UBE2S (+) N (%)	P value
Age			0.078
< 40	18 (50.0)	18 (50.0)	
40-60	35 (54.7)	29 (45.3)	
> 60	3 (21.4)	11 (78.6)	
Sex			0.869
Female	25 (50.0)	25 (50.0)	
Male	31 (48.4)	33 (51.6)	
Tumor stage			0.000
I-II	42 (77.8)	12 (22.2)	
III-IV	14 (23.3)	46 (76.7)	
Seizure			0.596
Absent	37 (47.4)	41 (52.6)	
Present	19 (52.8)	17 (47.2)	
IICP			0.194
Absent	27 (56.2)	21 (43.8)	
Present	29 (43.9)	37 (56.1)	
Cystic degeneration			0.562
Absent	45 (50.6)	44 (49.4)	
Present	11 (44.0)	14 (56.0)	
MTD			0.412
< 5 cm	37 (52.1)	34 (47.9)	
≥ 5 cm	19 (44.2)	24 (55.8)	
Chemoradiotherapy sensitivity			0.000
No	8 (13.1)	53 (86.9)	
Yes	48 (90.6)	5 (9.4)	

In summary, these findings show that UBE2S is highly expressed in grades III-IV glioma and the high expression level of UBE2S is correlated with glioma malignancy and chemo-radiotherapy resistance.

### UBE2S Is Associated With Worse Survival for Glioma Patients

Kaplan Meier survival curve was used to determine the prognostic value of UBE2S expression in the overall survival (OS) of glioma. In the TCGA cohort (n = 691), High UBE2S expression (> median value) was associated with poor prognosis whereas a low expression level of UBE2S was associated with good prognosis in LGG patients (p < 0.001, **Figures S2A, B**). However, UBE2S expression level was not associated with prognosis of GBM patients (p = 0.9157) in the TCGA cohort. In the CGGA cohort, high UBE2S expression level (> median value) was significantly associated with worse prognosis compared with low expression of UBE2S in LGG patients (p < 0.001), whereas this association was not observed in GBM patients (n = 325, p = 0.3924, **Figures S2C, D**). These findings show that high UBE2S expression was statistically correlated with shorter OS in LGG patients.

### UBE2S Acts Synergistically With Akt Phosphorylation in Promoting Glioblastoma Malignancy

**Figure 5:**
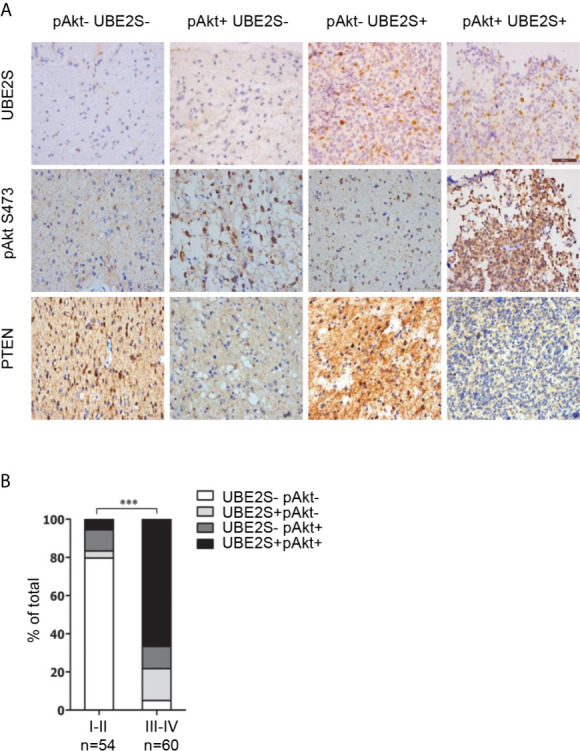
Association of UBE2S expression and activation of Akt pathway in glioma clinical samples. **(A)** Representative IHC staining for UBE2S, pAkt and PTEN on adjacent sections of formalin-fixed paraffin-embedded human glioma specimens. Scale bar: 50 μm. **(B)** Summary of the IHC assessment of UBE2S expression and Akt phosphorylation (pAkt) status in 114 human glioma patients. ***P < 0.001, based on χ2-test.

Abnormal expression of UBE2S in glioma prompted us to explore the clinical relevance of PI3K/Akt/UBE2S oncogenic synergy. Akt1 physically interacts with UBE2S and phosphorylates UBE2S at Thr152 in glioma cells, which is important for stabilization of UBE2S (14). To further validate this finding using clinical data, phosphorylation of Akt1 (S473, named pAkt) and UBE2S expression in 114 specimens were determined by immunohistochemistry (IHC) (**Figure 5A**). Percentage of UBE2S-expressing tumors (i.e., UBE2S+ pAkt- and UBE2S+ pAkt+) in grade III-IV tumors was significantly higher compared with that in grade II tumors (83.34% versus 9.26%; **Figure 5B** and **Table 2**). Significantly higher Akt phosphorylation positive specimens (UBE2S- pAkt+ and UBE2S+ pAkt+) were observed in grade III-IV group compared with grade II group (78.34% versus 16.67% in Grade II). Notably, a significant correlation was observed between UBE2S and phosphorylation of Akt1 (i.e. UBE2S+ pAkt+) and a worse pathological grade in Grade III-IV group compared with grade II group (Grade III-IV, 66.67%; Grade II, 5.56%; p < 0.001).

**Table 2:** Association of UBE2S and pAkt immunoreactivity with glioma.

Group	No. of specimens	Immunoreactivity			
		UBE2S- pAkt-	UBE2S+ pAkt-	UBE2S- pAkt+	UBE2S+ pAkt+
Grade I-II	n=54	43 (79.63%)	2 (3.70%)	6 (11.11%)	3 (5.56%)
Grade III-IV	n=60	3 (5%)	10 (16.67%)	7 (11.67%)	40(66.67%)
Total	n=114	46 (40.35%)	12 (10.53%)	13 (11.40%)	43 (37.72%)

In summary, these findings show that high UBE2S expression and active Akt1 are associated with adverse tumor characteristics. Therefore, PTEN-Akt1 pathways may positively regulate UBE2S function in glioma tissue by enhancing its stability.

### Mapping of Critical Lysine Residues for UBE2S Function

**Figure 6:**
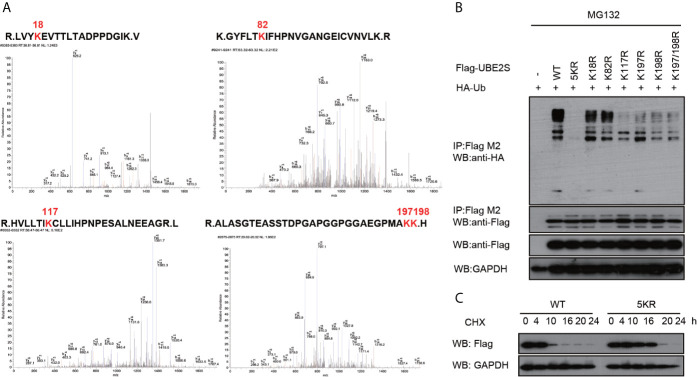
Validation of ubiquitination sites of UBE2S. **(A)** Identification of ubiquitination sites of UBE2S by *Mass Spectrometry*. **(B)** U87 cells were transfected with indicated plasmids and treated with 100 nM CHX for four hours before harvest. Representative pictures of whole-cell lysates and immunoprecipitants as analyzed by Western blotting using the indicated antibodies. Experiments were performed in triplicates. **(C)** U87 cells were transfected with indicated plasmids and treated with 100 nM CHX at different times as indicated. Representative pictures of UBE2S protein levels as analyzed by Western blotting. Experiments were performed in triplicates.

UBE2S is an unstable protein, and its degradation is dependent on a proteasome pathway, however, ubiquitination sites on this protein have not been explored. To explore UBE2S ubiquitination sites, U87 cells were transfected with Flag-UBE2S and the immunoprecipitated Flag-UBE2S was analyzed by mass spectrometry. Analysis showed that five lysine residues at position 18, 82, 117, 197 and 198 were ubiquitinated (**Figure 6A**). We hypothesized that these lysine ubiquitination sites may be required for UBE2S degradation in the nascent protein. The lysine residues were mutated to individually to arginine residues (K18R, K82R, K117R, K197R, and K198R), or as a group (named as UBE2S-5KR). The mutants were co-transfected with HA-Ubiquitin (HA-Ub) in U87 cells and ubiquitination of the mutants was analyzed by Western blot. UBE2S-5KR was less ubiquitinated compared with other individually mutants (**Figure 6B**). UBE2S-5KR was more stable in cycloheximide (CHX) treated cells compared with wild type UBE2S in U87 cells (**Figure 6C**).

### Knockdown of UBE2S Expression Increases GBM Sensitivity to Radiotherapy

**Figure 7:**
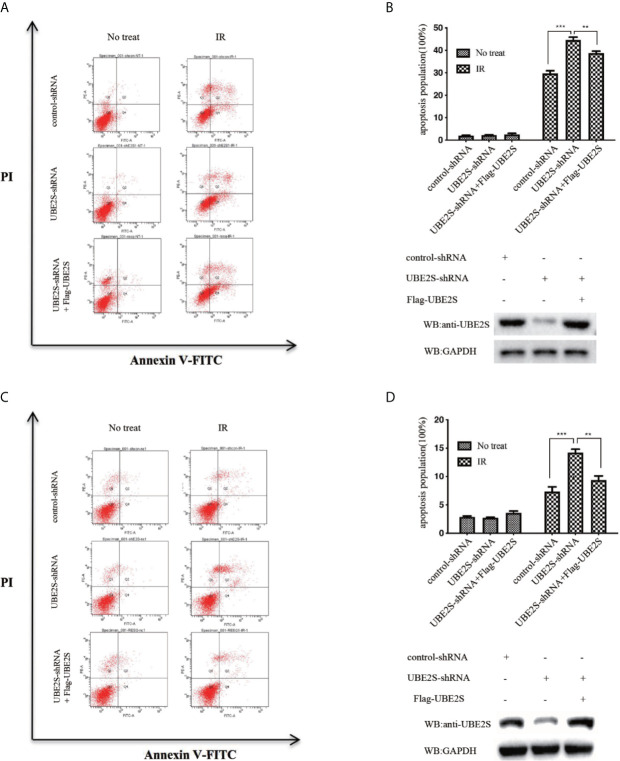
Knockdown of UBE2S expression increases sensitivity of U87 and U251 cells to IR-induced apoptosis. **(A)** Indicated cells were treated with IR, and apoptotic cells were analyzed using FACS. **(B)** The upper panel shows the graphical representation of FACS analysis in **(A)**. Results are derived from three independent experiments and presented as mean ± SEM. ***P < 0.001, **P < 0.01. The lower panel shows the protein expression levels as indicated. **(C)** Indicated cells were treated with IR, and apoptotic cells were analyzed using FACS. **(D)** The upper panel shows the graphical representation of the FACS analysis in **(C)**. Means represent three independent experiments. Error bars indicate SEM. ***P < 0.001, **P < 0.01. The lower panel shows the protein expression levels as indicated. Statistical differences were determined by ANOVA.

To explore the biological significance of UBE2S in radiotherapy resistance in glioma, U87 and U251 cells were exposed to 6MV X-ray and then the apoptosis rate was determined by flow cytometry. Knockdown of UBE2S expression increased sensitivity of U87 and U251 cells to IR-mediated apoptosis (**Figures 7A–D**). To explore the role of UBE2S in radiotherapy resistance, UBE2S expression was rescued in UBE2S knockdown cells. Cells transfected with Flag-tagged UBE2S showed significant decrease in apoptosis rate compared with the rate in the UBE2S knockdown group. Akt1 inhibitor MK-2206 reduces expression of UBE2S by reducing ubiquitination of UBE2S (14). To further explore the role of UBE2S in radiotherapy resistance, radiotherapy sensitivity of MK-2206 pre-treated glioma cells was determined by assaying apoptosis rate. MK-2206 pre-treated glioma cells were more sensitivity to IR compared with the control glioma cells (**Figures S3A–S3D**). In addition, MK-2206 pre-treated cells transfected with a plasmid expressing UBE2S-5KR effectively rescued apoptosis in these cells (**Figures S3B, 3D**). This finding shows that UBE2S overexpression is associated with radiotherapy resistance.

## Discussion

Glioma is the most common and most malignant intracranial tumor. It is characterized by high relapse rate and high mortality in adults. Despite advances in standard-of-care treatment, including surgical resection followed by radiotherapy and chemotherapy, the median overall survival of glioma patients has not significantly improved. Therefore, new therapeutic approaches are urgently needed.

UBE2S expression has been reported in various human cancers (8, 11, 15, 19), however its role in glioma is not known. In this work, 2 large datasets of glioma patients from the TCGA and the CGGA databases, as well as the clinical data from 114 glioma patients were used to perform a comprehensive analysis on the role of UBE2S on glioma. Analysis showed that UBE2S expression level was significantly up-regulated with increase in tumor grade in both TCGA and CGGA data set. The difference in UBE2S expression in molecular subtypes between TCGA and CGGA data set can be attributed to the different composition of patients in the two datasets. These results were further validated by IHC analysis, which showed high expression levels of protein in the GBM tissue compared with the level in the nonmalignant tissue. In this study, the expression level was positively correlated with the clinical stage of glioma, which is consistent with previous findings on correlation of UBE2S expression level with tumor grades in other types of tumors. These findings show that UBE2S expression level was significantly correlated with malignancy.

To explore the mechanism of action of UBE2S in participation of the above signaling pathways in glioma, association between UBE2S and the molecular genetic features of glioma in different grades was determined. Analysis showed that UBE2S expression was positively associated with PTEN-mutation and EGFR amplification and negatively associated to IDH1-mutation and co-deletion of 1p19q in whole grade glioma (**Figure 3**). In addition, UBE2S expression was positively correlated with PTEN-mutation and EGFR amplification, but negatively correlated with IDH1-mutation in LGG samples retrieved from TCGA dataset (**Figure 2**). However, no significant correlation was observed for GBM, which can be attributed to the heterogeneity of high-grade glioma. The positively correlated PTEN-mutation was also identified by IHC analysis, and its monoallelic mutations are mainly detected in glioblastoma (20). The mutation rate of PTEN was 30% - 40% in GBM, and these findings have had a significant impact on management of PTEN mutant subtypes of glioma. Moreover, studies report that EGFR amplification promotes invasion, proliferation and resistance to radiotherapy and chemotherapy in GBM (21–23). The negatively related IDH1-mutation in LGG and co-deletion of 1p19q in whole grade glioma, have been previously associated with prolonged PFS and OS in patients after treatment (24). Similarly, the findings of this study show that higher UBE2S expression level is correlated with a significantly poor overall survival in LGG. Uni- and multi-variate cox regression analysis showed that UBE2S was an independent prognostic marker for clinical outcome. Therefore, UBE2S is a vital biomarker for predicting prognosis of glioma.

UBE2S is involved in multiple processes and mediates development and progression of malignant tumors. Previous studies report that, UBE2S stabilizes hypoxia-inducible factor 1α (HIF-1α) by mediating proteosomal degradation of von Hippel-Lindau (VHL), and affects expression of Vascular Endothelial Growth Factor (VEGF) and induction of Epithelial Mesenchymal Transition (EMT) thus promoting cell proliferation, invasion and metastasis through pVHL-HIF pathway (9, 25, 26). Moreover, UBE2S knockdown reduces Tyr397 phosphorylation of Focal Adhesion Kinase (FAK) thus suppressing multiple signals for cell proliferation, migration, and invasion (13).

Pathway enrichment analyses showed that UBE2S is implicated in multiple biological processes, including G1/S transition of mitotic cell cycle, DNA replication, cell proliferation and DNA repair. These results established the critical character of UBE2S in tumor development. and Notably, these findings are consistent with existing findings on the major physiological function of UBE2S in elongation of K11 ubiquitin chains initiated by Anaphase-Promoting Complex (APC/C) and by promotion of degradation of APC/C substrates during mitosis and promoting mitotic exit (27).

In addition to contribution to tumor proliferation, UBE2S plays important roles in DNA repair. Radiotherapy with latest proton and carbon ion irradiation and chemotherapy with TMZ for GBM are associated with single and double-strand DNA breaks (28, 29). Therefore, key factors participating in DNA damage repair mechanism may be associated with chemo-radiotherapy resistance. Previous studies report that UBE2S knockdown increases sensitivity of cervical cancer HeLa cells to etoposide and doxorubicin. In addition, UBE2S knockdown increased chemosensitivity to topotecan, however, the mechanism is not known (8, 30). Furthermore, overexpression of UBE2S is significantly associated with poor response to neoadjuvant Computerized Controlled Radiation Therapy (CCRT) (30). In our previous study, UBE2S was associated with NHEJ-mediated DNA damage repair (14). Knockdown of UBE2S increases sensitivity of glioma cells to IR. Notably, rescue of UBE2S expression did not completely restore apoptosis inhibited by UBE2S knockdown, implying that additional molecular mechanisms are involved. Therefore, further studies should explore the mechanisms by which UBE2S promotes the apoptosis phenotype of glioma.

The unstable characteristic of UBE2S provide a basis for further exploration of its role in different tumors. Our previous study reported that increased stabilization of UBE2S occurs after phosphorylation by Akt1. Therefore, MK-2206, an Akt1 inhibitor, was used to explore the role of UBE2S in radiotherapy resistance. MK-2206 pre-treated glioma cells, which exhibited low UBE2S expression levels, were more sensitive to IR. However, re-expressed UBE2S-5KR, a stable mutant of UBE2S, effectively rescued apoptosis. Therefore, UBE2S is an effective predictor of chemo-radiotherapy sensitivity and MK-2206 may be used to reverse radiotherapy resistance by targeting UBE2S.

## Conclusions

In summary, the findings of this study show that higher levels of UBE2S are associated with a greater tumor burden, poor response to neoadjuvant therapy, and worse overall survival for LGG patients. This study shows the potential significance of UBE2S expression in diagnosis and prognosis of glioma and further explored the function of this protein in glioma. However, further studies are needed to confirm these results. The underlying mechanism provides a basis for UBE2S as a new molecular target for prevention and treatment of glioma. UBE2S is therefore a potential novel biomarker and therapeutic target for the treatment of human glioma.

## Data Availability Statement

The original contributions presented in the study are included in the article/**Supplementary Material**. Further inquiries can be directed to the corresponding author.

## Ethics Statement

The studies involving human participants were reviewed and approved by The Committees for the Ethical Review of Research at the First Affiliated Hospital of Harbin Medical University, Harbin, China. Written informed consent to participate in this study was provided by the participants’ legal guardian/next of kin. The animal study was reviewed and approved by The Committees for the Ethical Review of Research at the First Affiliated Hospital of Harbin Medical University, Harbin, China.

## Author Contributions

LH and XC performed all experiments, prepared figures and drafted the manuscript. LH, ZH, YY, SW, and YF participated in data analysis and interpretation of results. LH, CW, AW, and ZL designed the study and participated in data analysis. ZB and DR gave many valuable comments on the draft, and also polished it. All authors contributed to the article and approved the submitted version.

## Funding

This work was supported by National Natural Science Foundation of China (No. 81772678, 81802755, 81472360 and U20A20383), The China Postdoctoral Science Foundation (No. 2018M630372), Heilongjiang Postdoctoral Fund (No. LBH-Z17166), and Liaoning Science and Technology Plan Projects (No. 2011225034 to AW).

## Conflict of Interest

The authors declare that the research was conducted in the absence of any commercial or financial relationships that could be construed as a potential conflict of interest.

## Abbreviations

GBM, Glioblastoma multiform; TMZ, temozolomide; CGGA, Chinese Glioma Genome Atlas; TCGA, The Cancer Genome Atlas; VEGF, Vascular Endothelial Growth Factor; EMT, Epithelial Mesenchymal Transition; FAK, Focal Adhesion Kinase; CCRT, Computerized Controlled Radiation Therapy; GO, Gene ontology; KEGG, Kyoto encyclopedia of genes and genomes; OS, overall survival; LGG, Low-grade glioma; IR, ionizing radiation.
