# Supplementary material for: Molecular and Clinical Characterization of UBE2S in Glioma as a Biomarker for Poor Prognosis and Resistance to Chemo-Radiotherapy
Source: Front Oncol. 2021 May 27;11:640910. doi: 10.3389/fonc.2021.640910 (PMC8190380; doi:10.3389/fonc.2021.640910)
Supplement: Supplementary file 1 [file DataSheet_1.pdf]

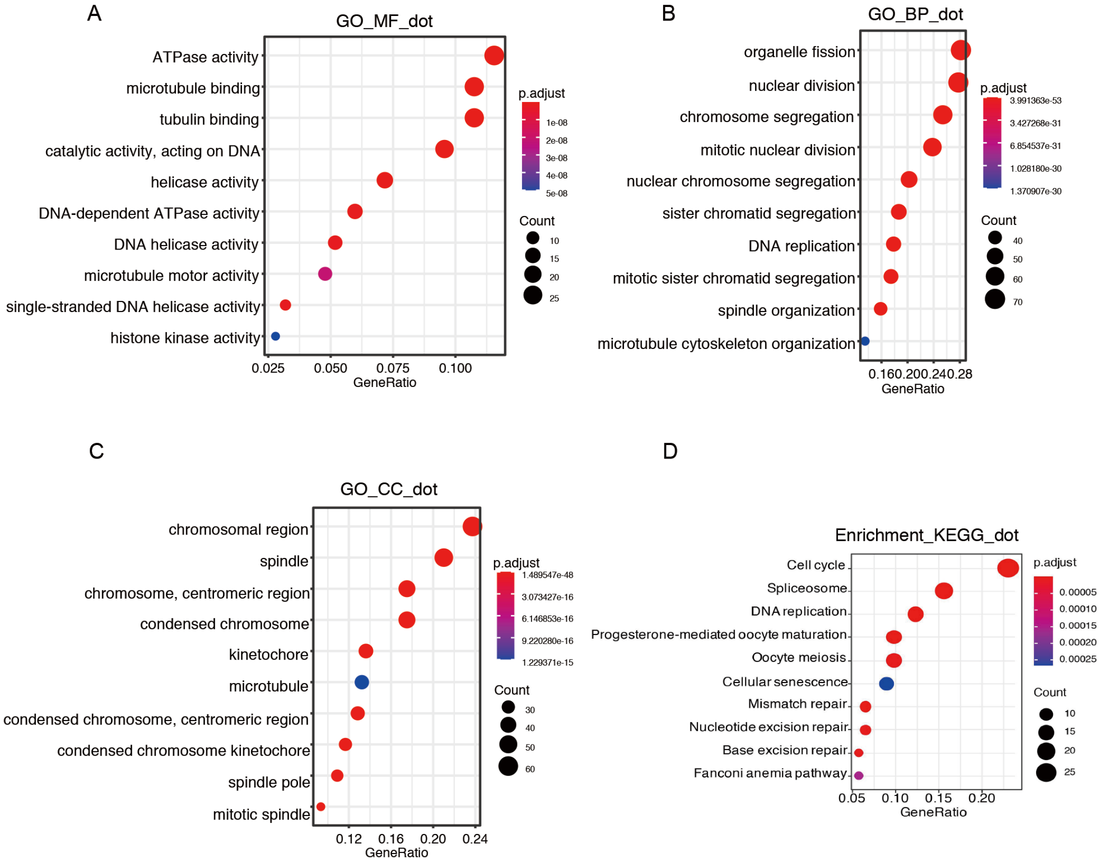

Figure S1. Gene ontology (GO) and Kyoto Encyclopedia of Genes and Genomes (KEGG) pathway analysis of UBE2S. (A) Enriched GO terms in the molecular function (MF). (B) Enriched GO terms in the biological process (BP). (C) Enriched GO terms in the cellular component (CC). (D) Enriched KEGG pathway associated with UBE2S.
