# Supplementary material for: Molecular and Clinical Characterization of UBE2S in Glioma as a Biomarker for Poor Prognosis and Resistance to Chemo-Radiotherapy
Source: Front Oncol. 2021 May 27;11:640910. doi: 10.3389/fonc.2021.640910 (PMC8190380; doi:10.3389/fonc.2021.640910)
Supplement: Supplementary file 2 [file DataSheet_2.pdf]

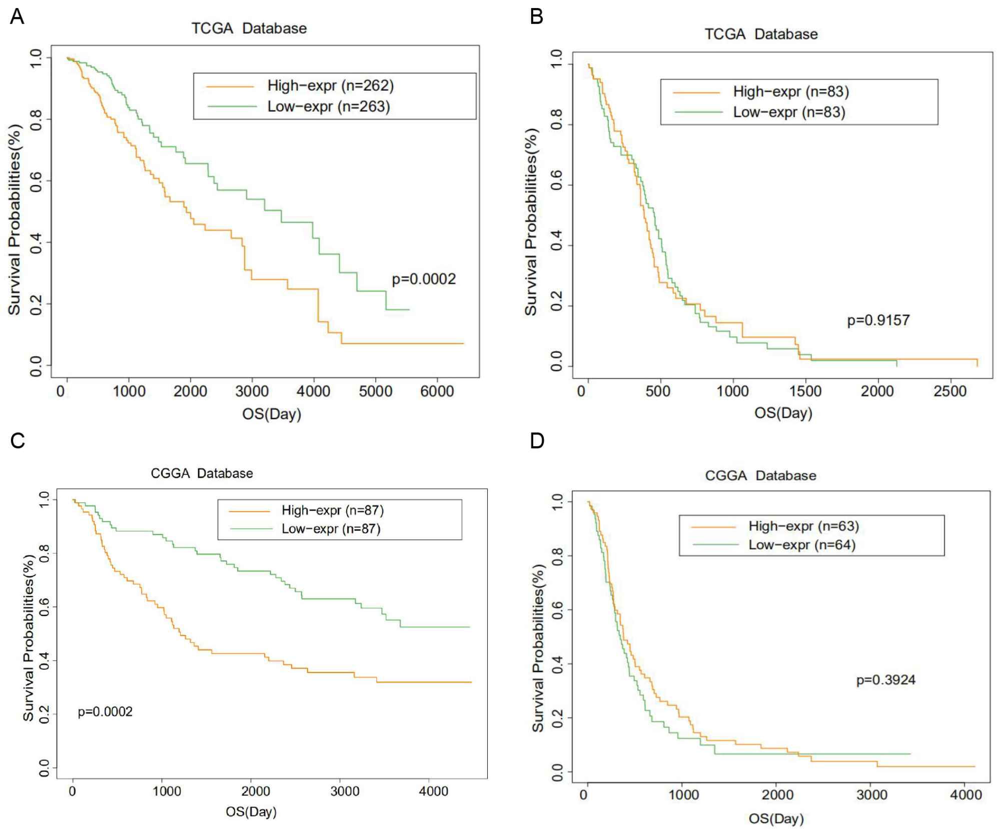

Figure S2. UBE2S is a prognostic factor in LGG glioma patients. (A, B) Kaplan-Meier survival analysis showed that high expression of UBE2S was significantly correlated with worse prognosis in LGG compared with GBM glioma patients in TCGA dataset. (C, D) Kaplan-Meier survival analysis showed that high expression of UBE2S was significantly correlated with worse prognosis in LGG compared with GBM glioma patients in CGGA dataset. P is based on log-rank test.
