# Supplementary material for: Molecular and Clinical Characterization of UBE2S in Glioma as a Biomarker for Poor Prognosis and Resistance to Chemo-Radiotherapy
Source: Front Oncol. 2021 May 27;11:640910. doi: 10.3389/fonc.2021.640910 (PMC8190380; doi:10.3389/fonc.2021.640910)
Supplement: Supplementary file 3 [file DataSheet_3.pdf]

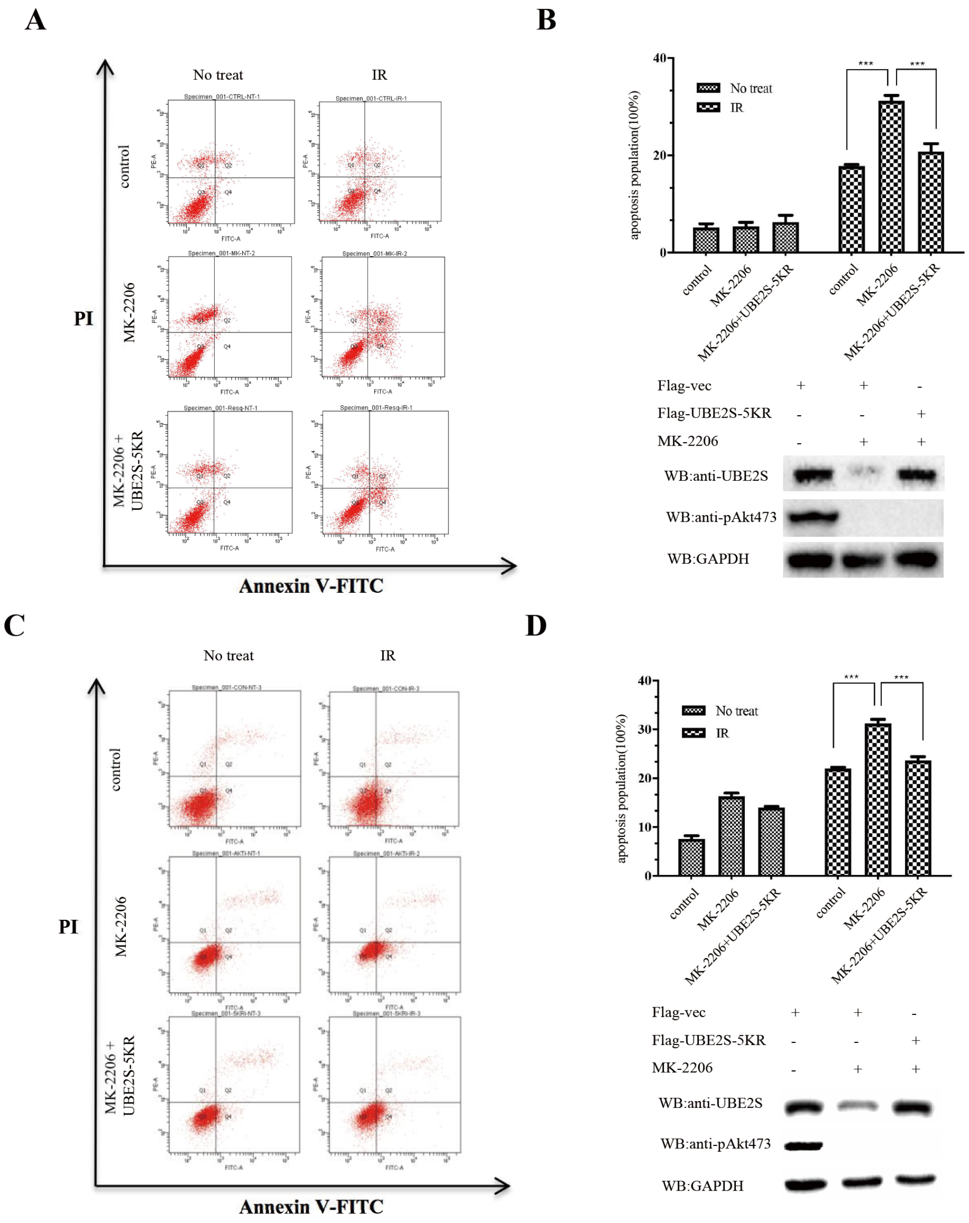

Figure S3. MK-2206 increases sensitivity of U87 and U251 cells to IR-induced apoptosis. (A) U87 cells were treated with Akt inhibitor MK-2206 as indicated, then followed by administration of IR and apoptotic cells were analyzed with FACS. (B) The upper panel shows the graphical representation of FACS analysis in (A). Results are derived from three independent experiments and presented as mean ± SEM. *** $P < 0.001$. The lower panel shows the protein expression levels as indicated. (C) U251 cells were treated with Akt inhibitor MK-2206 as indicated, followed by IR and apoptotic cells were analyzed with FACS. (D) The upper panel shows the graphical representation of FACS analysis in (C). Means represent three independent experiments. Error bars indicate SEM. *** $P < 0.001$. The lower panel shows the protein expression levels as indicated.
