# Supplementary material for: Molecular and Clinical Characterization of UBE2S in Glioma as a Biomarker for Poor Prognosis and Resistance to Chemo-Radiotherapy
Source: Front Oncol. 2021 May 27;11:640910. doi: 10.3389/fonc.2021.640910 (PMC8190380; doi:10.3389/fonc.2021.640910)
Supplement: Supplementary file 4 [file Table_1.docx]

Table S1. Univariate and Multivariate Analyses of Factors Associated with the chemoradiotherapy sensitivity of glioma patients (n=114).

| Variables | | **Univariate analysis** | | | | | | | **Multivariate analysis** | | | | |
| --- | --- | --- | --- | --- | --- | --- | --- | --- | --- | --- | --- | --- | --- |
|  |  | HR | | 95% CI | | P | | | HR | | 95% CI | | P |
| Expression | High vs. Low | | 0.016 | | 0.005-0.051 | | 0.000* | 0.012 | | 0.002-0.069 | | 0.000* | |
| Age | ≥ 60 years vs. < 60 years | | 0.371 | | 0.111-1.245 | | 0.108 | 1.220 | | 0.142-10.510 | | 0.856 | |
| Sex | Male vs. Female | | 0.583 | | 0.276-1.230 | | 0.157 | 0.393 | | 0.083-1.872 | | 0.241 | |
| Tumor stage | III-IV vs. I-II | | 0.064 | | 0.026-0.160 | | 0.000* | 0.105 | | 0.023-0.483 | | 0.004* | |
| Seizure | Present vs. Absent | | 0.886 | | 0.401-1.960 | | 0.766 | 0.455 | | 0.096-2.158 | | 0.322 | |
| IICP | Present vs. Absent | | 0.586 | | 0.277-1.241 | | 0.162 | 0.934 | | 0.214-4.066 | | 0.927 | |
| Cystic degeneration | Present vs. Absent | | 0.879 | | 0.360-2.147 | | 0.778 | 1.876 | | 0.340-10.347 | | 0.470 | |
| MTD | ≥ 5 cm vs. < 5 cm | | 1.001 | | 0.469-2.140 | | 0.997 | 3.842 | | 0.712-20.739 | | 0.118 | |
